# Surface engineered mesoporous silica carriers for the controlled delivery of anticancer drug 5-fluorouracil: Computational approach for the drug-carrier interactions using density functional theory

**DOI:** 10.3389/fphar.2023.1146562

**Published:** 2023-04-13

**Authors:** Fozia Rehman, Asif Jamal Khan, Zaib Us Sama, Hussah M. Alobaid, Mazhar Amjad Gilani, Sher Zaman Safi, Nawshad Muhammad, Abdur Rahim, Abid Ali, Jiahua Guo, Muhammad Arshad, Talha Bin Emran

**Affiliations:** ^1^ Interdisciplinary Research Centre in Biomedical Materials (IRCBM), COMSATS University Islamabad, Lahore Campus, Lahore, Pakistan; ^2^ Institute of Chemistry, University of Campinas, UNICAMP, Campinas, São Paulo, Brazil; ^3^ College of Urban and Environmental Sciences, Northwest University, Xi’an, Shaanxi, China; ^4^ Department of Chemistry, Islamia College, University of Peshawar, Peshawar, Pakistan; ^5^ Department of Zoology, College of Science, King Saud University, Riyadh, Saudi Arabia; ^6^ Department of Chemistry, COMSATS University Islamabad, Lahore Campus, Islamabad, Pakistan; ^7^ Faculty of Medicine, Bioscience and Nursing, MAHSA University, Jenjarom, Selangor, Malaysia; ^8^ Institute of Basic Medical Sciences, Khyber Medical University, Peshawar, Pakistan; ^9^ Department of Chemistry, COMSATS University Islamabad, Islamabad, Pakistan; ^10^ Department of Zoology, Abdul Wali Khan University, Mardan, Pakistan; ^11^ Shaanxi Key Laboratory of Earth Surface System and Environmental Carrying Capacity, College of Urban and Environmental Sciences, Northwest University, Xi’an, China; ^12^ Jhang Campus, University of Veterinary and Animal Sciences, Lahore, Pakistan; ^13^ Department of Pharmacy, BGC Trust University Bangladesh, Chittagong, Bangladesh; ^14^ Department of Pharmacy, Faculty of Allied Health Sciences, Daffodil International University, Dhaka, Bangladesh

**Keywords:** surface modification, SBA-15, 5-fluorouracil, drug–carrier interactions, anti-cancer, DFT

## Abstract

**Introduction:** Drug delivery systems are the topmost priority to increase drug safety and efficacy. In this study, hybrid porous silicates SBA-15 and its derivatives SBA@N and SBA@3N were synthesized and loaded with an anticancer drug, 5-fluorouracil. The drug release was studied in a simulated physiological environment.

**Method:** These materials were characterized for their textural and physio-chemical properties by scanning electron microscopy (SEM), nuclear magnetic resonance (NMR), Fourier transform infrared spectroscopy (FTIR), small-angle X-ray diffraction (SAX), and nitrogen adsorption/desorption techniques. The surface electrostatics of the materials was measured by zeta potential.

**Results:** The drug loading efficiency of the prepared hybrid materials was about 10%. *In vitro* drug release profiles were obtained in simulated fluids. Slow drug release kinetics was observed for SBA@3N, which released 7.5% of the entrapped drug in simulated intestinal fluid (SIF, pH 7.2) and 33% in simulated body fluid (SBF, pH 7.2) for 72 h. The material SBA@N presented an initial burst release of 13% in simulated intestinal fluid and 32.6% in simulated gastric fluid (SGF, pH 1.2), while about 70% of the drug was released within the next 72 h. Density functional theory (DFT) calculations have also supported the slow drug release from the SBA@3N material. The release mechanism of the drug from the prepared carriers was studied by first-order, second-order, Korsmeyer–Peppas, Hixson–Crowell, and Higuchi kinetic models. The drug release from these carriers follows Fickian diffusion and zero-order kinetics in SGF and SBF, whereas first-order, non-Fickian diffusion, and case-II transport were observed in SIF.

**Discussion:** Based on these findings, the proposed synthesized hybrid materials may be suggested as a potential drug delivery system for anti-cancer drugs such as 5-fluorouracil.

## Graphical Abstract

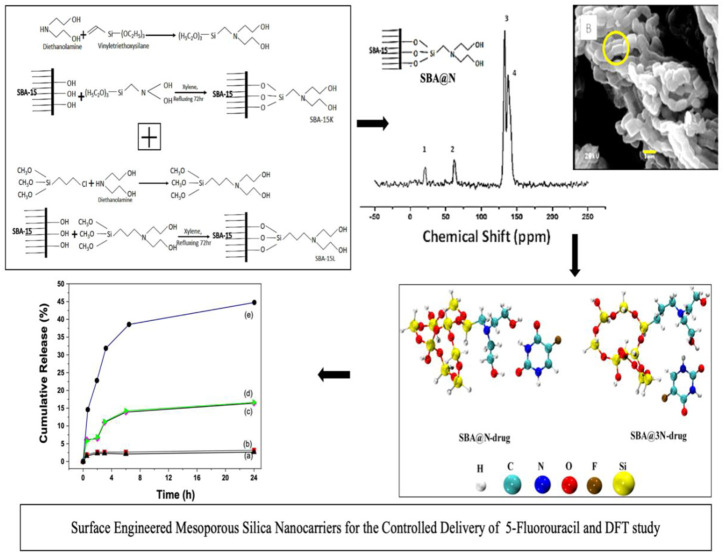

## 1 Introduction

Cancer is an increasing threat to human life and health and is the second leading cause of death. Therefore, the development of safe and effective treatments against this disease is very important (Fadeel and Alexiou, 2020). Chemotherapy and radiotherapy are the most common treatment options for cancer patients. Despite advancements in cancer treatment, administration of conventional anticancer therapeutics remains the first-line therapeutic option (Benedicto et al., 2021). The major disadvantages of direct chemotherapy delivery are their low therapeutic index and low bioavailability, non-specific targeting, need for high doses, and the most common development of multiple drug resistance. Consequently, new drug delivery systems are the top priority to address these issues to reduce the undesirable side effects of chemotherapy (Russell-Jones et al., 2004; Arias, 2008; Chandran et al., 2017).

5-Fluorouracil (5-FU) is a heterocyclic aromatic compound, similar in structure to the pyrimidine molecules of deoxyribonucleic acid (DNA) and ribonucleic acid (RNA) (analog of uracil with a fluorine atom at the carbon 5 in the aromatic ring). 5-FU is the first rationally synthesized anticancer drug approved by the U.S. Food and Drug Administration (FDA) to treat multiple solid tumors (Longley et al., 2003). This drug is administered as an injection to treat a range of conditions, including gastric, rectal, colon, liver, pancreatic, ovarian, bladder, and breast cancers. This drug is applied topically (cream) to treat actinic keratosis (a skin condition that may become cancerous) and certain types of basal cell skin cancer (Sharma et al., 2012; Chandran et al., 2017). This drug acts by interfering with nucleoside metabolism and thus can be incorporated into DNA and RNA, leading to cytotoxicity and later cell death (Longley et al., 2003; Sara et al., 2018).

Currently, 5-FU is being administered in combination with various other chemotherapeutic drugs. However, the high dose, reduced bioavailability, shorter half-life, and lower absorption are the serious drawbacks of these oral formulations. Furthermore, according to the FDA, 5-FU has a wide range of serious side effects, including neurologic toxicity, hyperammonemic encephalopathy, myelosuppression, mucositis, and cardiotoxicity. In basic intestinal media, this drug also produces byproducts that are toxic to the heart (Longley et al., 2003; Shoemaker et al., 2004; Sara et al., 2018). Due to its short half-life and fast metabolism, 5-FU is administered intravenously IV), in which maintenance of drug concentration in the blood is achieved through frequent doses. This drug is metabolized in the gastrointestinal tract when administered orally. Considering these shortcomings, a controlled and targeted oral drug delivery to the desired site of action is required to enhance the efficacy and reduce the cytotoxicity to the normal cell. The development of new carrier systems in nanometric size is required to tackle the associated problems with the current anticancer drugs.

The main aim of designing a drug carrier system is to address the limitations related to drug delivery to the desired sites of therapeutic action while minimizing the adverse side effects and managing the therapeutics with great selectivity and control of the targeted sites (Davis et al., 2008; Cal et al., 2014). For this purpose, so far, many materials, e.g., polymers, ceramics, bioactive glasses, organic–inorganic materials, proteins, and lipids, have been reported (Vallet-Regí, 2006; Iqbal et al., 2019; Khan et al., 2019; Tayyab et al., 2019; Malik et al., 2020; Pasha et al., 2020; Fuloria et al., 2022; Karthic et al., 2022; Nawaz et al., 2022; Thalluri et al., 2022).

Among these materials, hydrophobic polymer nanocapsules (nanolipid capsules), micelles, and hydrogels can easily solubilize water-soluble drugs into their structures and encapsulate them; however, these materials are physiochemically unstable, which results in unwanted immature drug leakage (Cal et al., 2014).

Mesoporous silica is biocompatible and physiochemically and biochemically stable compared to other inorganic materials (Fuloria et al., 2022; Karthic et al., 2022; Thalluri et al., 2022). Other advantages of these silica materials are surface modification, the ability of tunable pore size, pore volume, and high surface area, which make these materials suitable for many applications (Vallet-Regi et al., 2001; Vallet-Regí et al., 2006; He et al., 2011; Lin et al., 2011; Rehman et al., 2014a; Boccardi et al., 2015). Currently, these materials have attracted enormous attention for a variety of biomedical applications (Ahmed et al., 2016; Rehman et al., 2018; Rauti et al., 2019). Due to their biocompatible nature and high drug loading capacity, mesoporous silica has attracted significant attention in drug delivery (Vallet-Regí et al., 2006; He et al., 2011; Lin et al., 2011; Rehman et al., 2014a). Furthermore, the porous stable network (to safeguard small molecules from harsh environment) and the surface modification of these materials with organic functional groups (which allows great control over drug release) make these materials an ideal option for controlled and targeted drug delivery (Yuan et al., 2011; Kango et al., 2013; Amolegbe et al., 2016). The recent advancements in nanomedicines and bioengineering are changing the future of drug development and diagnostics.

A breakthrough in therapeutics has introduced new drugs in healthcare; however, they still face the setbacks of therapy resistance and lack of response, as well as suffering from serious adverse effects. Therefore, devising new therapeutic strategies and exploring innovative treatment combinations are the major areas of focus (Kruk et al., 2000).

This work aimed to synthesize surface-modified silica type SBA-15 to overcome the current issues of drug delivery systems of 5-FU, such as instability in the physiochemical environment, undesired immature leakage before reaching the target site, and controlled release of drug molecules. For this purpose, SBA-15 and its derivatives SBA@N and SBA@3N were synthesized, characterized, and applied for loading and release of 5-FU in a simulated physiological environment. A density functional theory (DFT) study was conducted to determine the drug–carrier interactions. The strong interaction of the 5-FU drug with the prepared carriers, and hence its slow release, has been supported by the interaction energies calculated from DFT simulations.

## 2 Experimental

### 2.1 Materials and methods

Analytical-grade ethanol, xylene, hydrochloric acid (HCl), sodium chloride, sodium hydroxide, Tris (hydroxymethyl) aminomethane (NH_2_C (CH_2_OH)_3_, potassium chloride, sodium bicarbonate, magnesium chloride hexahydrate, sodium sulfate, calcium chloride, potassium phosphate, dibasic trihydrate (K_2_HPO_4_·3H_2_O), sodium carbonate, tetraethylorthosilicate (TEOS), 5-fluorouracil(5-FU), diethanolamine, vinyltriethoxysilane, and Pluronic P123 obtained from Sigma Aldrich were used in this work. Deionized water was used during the experiments.

#### 2.1.1 SBA-15 synthesis

SBA-15 silica was synthesized as previously reported (Zhao et al., 1979). To obtain 1 g of silica, about 2.0 g of the structure-directing agent Pluronic P123 triblock polymer was stirred in deionized water (12.0 cm^3^) and HCl solution (60.0 cm^3^, 2.0 mol dm (Russell-Jones et al., 2004)) at 313 K at a speed of 200 rpm (revolution per minute) until the mixture became homogenous. About 4.0 g of TEOS was added dropwise to the reaction container and further stirred for 4 h. Afterward, the resulting product was autoclaved for crystallization for 24 h at 373 K. The prepared SBA-15 was then filtered and washed with deionized water to get a neutral pH). The powder was dried at room temperature for 48 h. To remove the surfactant P123, calcination was performed at 873 K for 6 h at a heating ramp rate of 2 per minute (Song et al., 2005; Fagundes et al., 2006).

**FIGURE 1:**
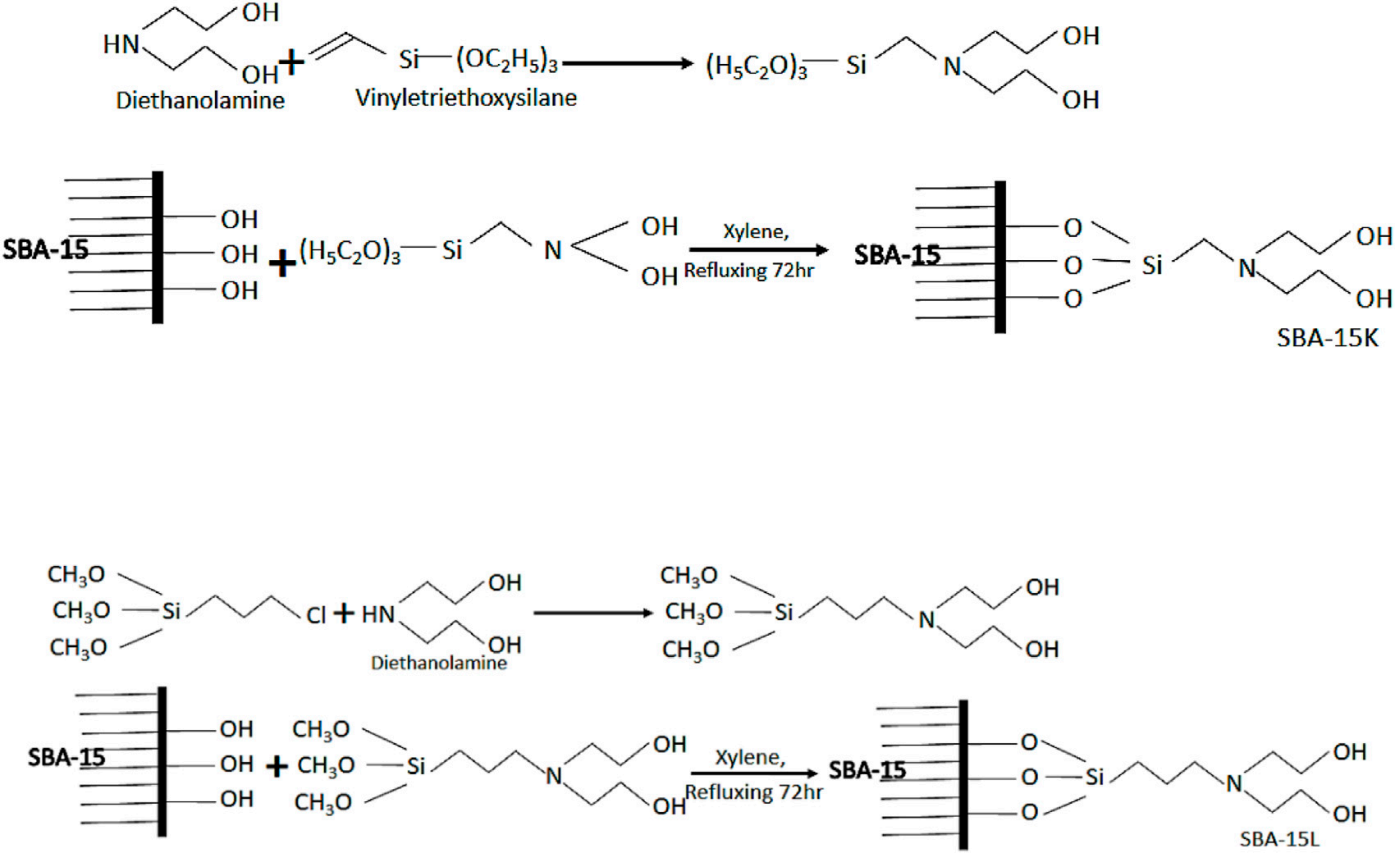
Schematic representation for the fabrication of silica SBA@N (Scheme 1) and SBA@3N (Scheme 2).

To obtain surface-modified silica SBA@N, about 37.0 mmol of vinyltriethoxysilane was added with diethanolamine (37.0 mmol) in 70 cm^3^ of ethanol at 323 K for 72 h to get a new silylating agent. This synthesized novel silylating agent (Scheme 1) was then reacted with the surface silanols of SBA-15 (1.0 g) dispersed in xylene. This process was performed for 72 h at 348 K in a dry nitrogen environment. The final product SBA@N was then filtered and washed to a neutral pH and dried under a vacuum at room temperature. Another material, SBA@3N, was synthesized using the same procedure as aforementioned. For synthesizing this material, in step 1, 3-chloropropyltrimethoxysilane (37.0 mmol) was reacted with diethanolamine (18.0 mmol) in 70 cm^3^ of ethanol at 323 K for 72 h to get a new silylating agent. For surface modification, about 1.0 g of SBA-15 was reacted with the obtained product in step 1. The solid silica was purified, cleaned, and incubated at room temperature in air. The schematic diagrams are shown in Figure 1.

#### 2.1.2 Drug loading and release

Drug loading and release tests were conducted using the immersion method. In two separate containers, 0.5 g of silica was soaked in 50 cm^3^ (10 g dm^−3^) of the 5-FU solution prepared in deionized water. To prevent evaporation, these containers were closed, and the samples were incubated for 72 h and then filtered. From each filtrate, 3.0 cm^3^ was diluted to a further 50.0 cm^3^ and was analyzed with a UV–Vis spectrophotometer. Eq. 1 was used to compute the drug’s loading capacity (wt/wt percent) (Rehman et al., 2016; Rehman et al., 2018).

$$wt/wt\%=\frac{m_{1}-\frac{50}{v}CV}{m_{2}+\left(m_{1}-\frac{50}{v}\;CV\right)}100,$$ (1)

where m_1_ is the mass of the drug, m_2_ is the mass of silica, C is the drug concentration in the filtrate diluted to 50 cm^3^, v is the filtrate volume, and V is the solvent volume.

*In vitro* drug release profiles were studied in simulated (gastric fluid (SGF/0.1 M HCl; pH 1.2; USP; without pepsin), intestinal fluid (SIF/phosphate buffer without pancreatin; pH 7.2; USP 26), and body fluid (SBF; pH 6.8; USP). These fluids were prepared by dissolving 0.305 g MgCl_2_·6H_2_O; 0.350 g NaHCO_3_; 0.224 g of KCL; 7.996 g NaCl; 40 cm^3^ HCl (1.0 M); 0.278 g CaCl_2_; 0.071 g Na_2_SO_4,_ and 6.057 g Tris (hydroxymethyl) aminomethane (NH_2_C(CH_2_OH) _3_ in 1 L of deionized water. For the drug release studies, each 50.0 mg of the drug-loaded samples (pressed tablets at 5.0 MPa) was soaked in 250 cm^3^ of the release medium. The sample and separate (SS) method was used to measure the released drug concentration. To analyze the drug release from a soaked tablet, 3.0 cm^3^ of the sample was collected at intervals of 0.5, 1.0, 3.5, 7.0, 8.5, 21.0, 24.0, and 72.5 h.

The drug concentration was determined using a Shimadzu MultiSpec-1501 spectrophotometer. The original volume of the release medium was maintained by adding 3.0 cm^3^ of fresh medium each time. A correction method was applied to the release data (Rana et al., 2011). All the analyses were performed in triplicate.

#### 2.1.3 Computational methodology

The choice of a representative model for SBA-15 silica is crucial in conducting the theoretical studies. The previous studies have proved that the bicyclic 5–6s cluster model is reliable in interpreting the properties of SBA-15 silica (Wang et al., 2011) and its subsequent derivatives (Laskowski and Laskowska, 2014). Therefore, we have chosen the same bicyclic 5–6s cluster model to represent the prepared derivatives of SBA-15 (SBA@N and SBA@3N). All the structures have been optimized at B3LYP (Zhao et al., 1979; Yuan et al., 2011; Authors Anonymous, 2020) functional with a 6-31G(d,p) basis set. The frequency calculations have been performed at the same level of theory to ascertain that the optimized structures are true minima.

To confirm the interaction of the 5-fluorouracil drug with the functionalized SBA-15, adsorption energies have been calculated by using Eq. 2:

$$E_{ads}=E_{functionalized\;SBA15-drug}-\left(E_{functionalized\;SBA15}+E_{drug}\right).$$ (2)

Here, $E_{functionalized\;SBA15-drug}$ is the total energy of functionalized SBA-15 containing the drug, $E_{functionalized\;SBA15}$ is the energy for pure functionalized SBA-15, and $E_{drug}$ is the optimized drug energy, respectively.

All the DFT computations were executed using the Gaussian 16 program package (Zhao et al., 1979; Fagundes et al., 2006), and the geometries were visualized by GaussView 6.1.1 software (Frisch et al., 2016; Sharif et al., 2022). The optimized structures have been drawn using VMD software (Wu et al., 2020).

## 3 Characterizations

Quantitative analysis of the prepared samples was performed with the PerkinElmer PE-2400 instrument to check the elemental composition.

A Bruker Avance 300 MHz spectrometer in solid-state was used to obtain the NMR spectra at room temperature. Each sample was prepared by compacting 1 g of silica in 4 mm ZrO_2_ rotors. The data were recorded at 59.63 MHz and 75.47 MHz ( magic-angle spinning of 10 MHz), with a 3-s pulse repetition time and a 4-millisecond contact time.

Micromeritics ASAP 2000 Quanta Chrome Autosorb was used for nitrogen adsorption/desorption. Samples were degassed before the analysis at 363 K for 8 h.

A Fourier transform infrared (FTIR) spectrophotometer, model Bomem MB-series, was used to collect the FTIR spectra. Sample scans were performed using KBr pellets. The spectra were acquired with a total of 32 scans and a resolution of 4 cm^-1^ in the wavelength range 400 to 4,000 cm^−1^.

The crystalline structure of the produced samples was examined using the small-angle X-ray crystallography method (SAXs) using a synchrotron light D11A-SAXS line with a wavelength of 0.1488 nm.

SEM micrographs were taken with a JEOL JS 6360-LV scanning electron microscope. The Nano-ZS Zetasizer was used to measure the zeta potential. Silica samples were suspended in KCL solution for the measurement.

The *in vitro drug* release experiments were performed in a temperature-controlled shaker-incubator model MA-420-MARCONI. The drug concentration was measured with a Shimadzu-MultiSpec-1501 spectrophotometer.

A centrifuge model, Hettich Rotina-38 Zentrifugen, was used to centrifuge the drug/silica suspension at a rate of 4,000 rpm, and the SevenEasy Mettler-Toledo pH meter was used to check the pH.

## 4 Results and discussion

### 4.1 Elemental analysis

The modified silica samples were analyzed for carbon and nitrogen contents using the elemental analyzer. The percent content of carbon and nitrogen is given in Table 1. The carbon contents of SBA@N and SBA@3N were found to be 6.37% and 7.55% and nitrogen content 0.28% and 0.60%, respectively. The degree of functionality (δ) was calculated with Eq. 3. The δ values (based on carbon %) were found to be 5.30 mmol g^−1^ and 6.30 mmol g^−1^ for SBA@N and SBA@3N, respectively.

$$\delta =\frac{E\%}{m_{a}}\times 10,$$ (3)

where $m_{a}$ represents an element’s atomic mass and *E%* is the element percentage obtained through the elemental analyzer.

**TABLE 1:** Elemental analysis data: carbon and nitrogen contents (C% and N%), degree of functionality (δ/mmolg^−1^), surface area (S_BET_), pore volume (V_p_), and pore diameter (D_p_) obtained with nitrogen adsorption and desorption techniques.

Silica	C%	N%	δ/mmolg^−1^	S_BET_/m^2^g^−1^	V_p_/cm^3^g^−1^	D_p_/nm
SBA@N	6.37	0.28	5.30	310.85	0.51	6.82
SBA@3N	7.55	0.60	6.3	457.86	0.92	5.21

### 4.2 Nitrogen adsorption/desorption

**FIGURE 2:**
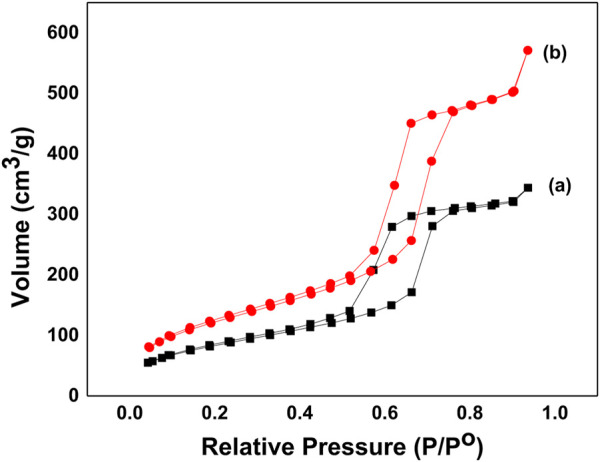
Nitrogen adsorption/desorption graphs for (a) SBA@N and (b) SBA@3N.

The nitrogen adsorption/desorption approach was used to determine the main structural features of the manufactured materials, like surface area, pore size, and diameter. Typical type IV adsorption/desorption isotherms were observed for modified materials SBA@N and SBA@3N (Figure 2), which is a typical characteristic of mesoporous type materials as reported previously (Rehman et al., 2014b). These isotherms also suggest that the original mesoporous structure of SBA-15 silica remained preserved during the calcination and surface modification processes (Rehman et al., 2016). The surface area was calculated using the Brunauer, Emmett, and Teller (BET) method, which is widely used to calculate the contact area of mesoporous silica through the adsorption process of gas molecules. Eq. 4 can be used to evaluate the surface area model of the BET.

$$S_{BET}=\frac{V_{m}N.a}{m24400}$$ (4)

where Avogadro’s number is N (6.022 × 1023 mol^−1^). For nitrogen, a represents the cross-sectional area of the gas molecules, which is 0.162 nm^2^, and the mass of the sample in the sample holder is m. Also, for temperature and atmospheric pressure, 22,400 is the optimum volume of a gas mole (TPP) in cm^3^.

After surface modification, the BET surface area of both materials decreased as compared to that of the parent unmodified material, SBA-15 (1,071.0 m^2^g^−1^). For SBA@N and SBA@3N materials, the BET surface area was found to be 310.85 m^2^g^-1^ and 457.85 m^2^ g^−1^, respectively. The mean pore diameter, size, and volume distribution from nitrogen sorption isotherms were determined using the BJH (Barrett, Joyner, and Halenda) method. The concept of capillary condensation underpins the primary assumptions for estimating pore diameters. The adsorptive will condense in the apertures when critical pressure is attained. As a result, if condensation can happen at a given pressure, the pore radius can be computed. From the physisorption data, this is observed as a hysteresis loop (Figure 2). Eq. 5 gives the Kelvin radius (r_Ks_) based on applied pressure for capillary condensation in cylindrical holes.

$$r_{K}\left(\frac{p}{p^{0}}\right)=\frac{2\gamma V_{L}}{RTIn\frac{p}{p^{0}}},$$ (5)

where P and P^o^ indicate the adsorbate’s optimum and saturation pressures, respectively; γ is the absorbed liquid’s surface tension; V_L_ is the liquid’s molecular volume; and *r*_*K*_ is the mean radius of the liquid meniscus. The pore size, *r*_*P*_, is then derived by combining the thickness of the adsorbed layer, *t*, to *r*_*K*_ (Eq. 6).

$$r_{p}=2\left(r_{K}+t\right).$$ (6)

The difference in the amount of the adsorptive for each step in the isotherm shows that the core volume was filled or the depleted form of all pores is homogeneous to determine the number of pores of this size. Pores of this radius have a total length as well as the area of these pores, which may be determined using the volume difference between the core and the radius of a cylinder r_P_. The overall pore distribution can be derived by conducting similar computations for all steps in the isotherms.

The pore volume (BJH) was reduced after the modification of SBA-15 channels. For SBA@N, this value changed from 1.98 cm^3^g^−1^ to 0.51 cm^3^g^−1^ and to 0.92 cm^3^g^−1^ for the SBA@3N material. The pore diameter value is listed in Table 1. The observed changes in the structural parameters suggested the successful modification of SBA-15 (Galarneau et al., 2001).

### 4.3 Fourier transform infrared spectroscopy

**FIGURE 3:**
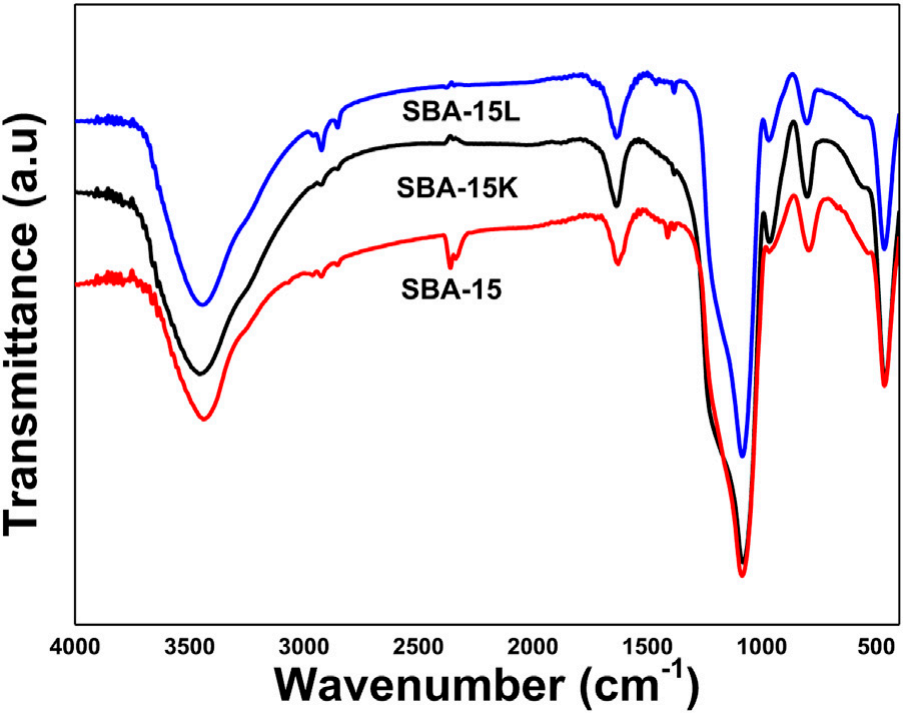
SBA-15, SBA@N, and SBA@3N silica’s FTIR spectra.

The FTIR spectra of SBA-15 and its derivatives, SBA-15 and SBA@3N, are shown in Figure 3. The broad bands that appeared for all materials in the range 3,000–3,500 cm^−1^ were attributed to the stretching vibrations of the silanol groups on the silica surface and the OH groups of the trapped H_2_O molecules inside the porous channels, as well as to the attached diethanolamine groups, while the band in 2,800 to 2931 cm^−1^ can be assigned to ν (C–H) stretching. Those bands that appeared around 1,000 to 1,100 cm^−1^ intervals represent the Si–O–Si stretching vibration. Bands for surface silanol groups (Si-OH) appeared at 792 cm^−1^. New bands are expected to appear when the surface silanol groups are replaced by the silylating agent. For the modified materials, SBA@N and SBA@3N bands that appeared in the region 1,398 to 1,469 cm^−1^ can be assigned to the C–N stretching vibration of diethanolamine groups adhered on the surface of the silica.

### 4.4 ^13^C NMR CP-MAS spectroscopy

**FIGURE 4:**
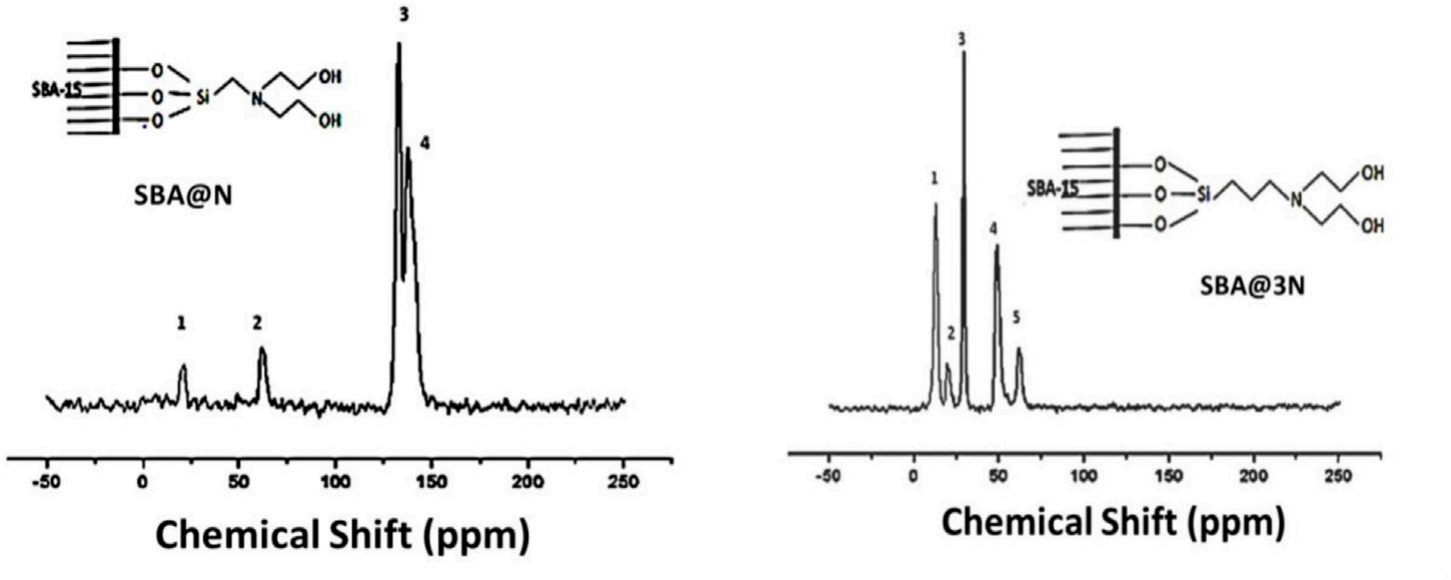
^13^C NMR CP-MAS spectra of SBA@N and SBA@3N materials.

Figure 4 shows the ^13^C NMR spectra of the prepared materials. The spectra of SBA@N showed chemical shifts, appearing around 23.0 and 70.0 ppm, which can be assigned to Si-C, C-C, C-N, and C-O bonds. Two major chemical shifts between 125 and 150 ppm can be assigned to the entrapped solvent in the mesopores of this silica (Wang et al., 2005; Rehman et al., 2014a; Khan et al., 2020) (Scheme 1). Similarly, the chemical shifts that appeared in the spectrum of SBA@3N around 10–25 ppm can be assigned to Si-C and C-C linkages, while signals around 50 ppm can be assigned to C-N. The chemical shift around 65 ppm can be assigned to C-O bonds (Scheme 2). The appearance of these chemical shifts (Figure 4) confirmed the modification of the silica surface with the prepared silylating agents containing diethanolamine functional groups.

### 4.5 Small-angle X-ray (SAX) diffraction analysis

**FIGURE 5:**
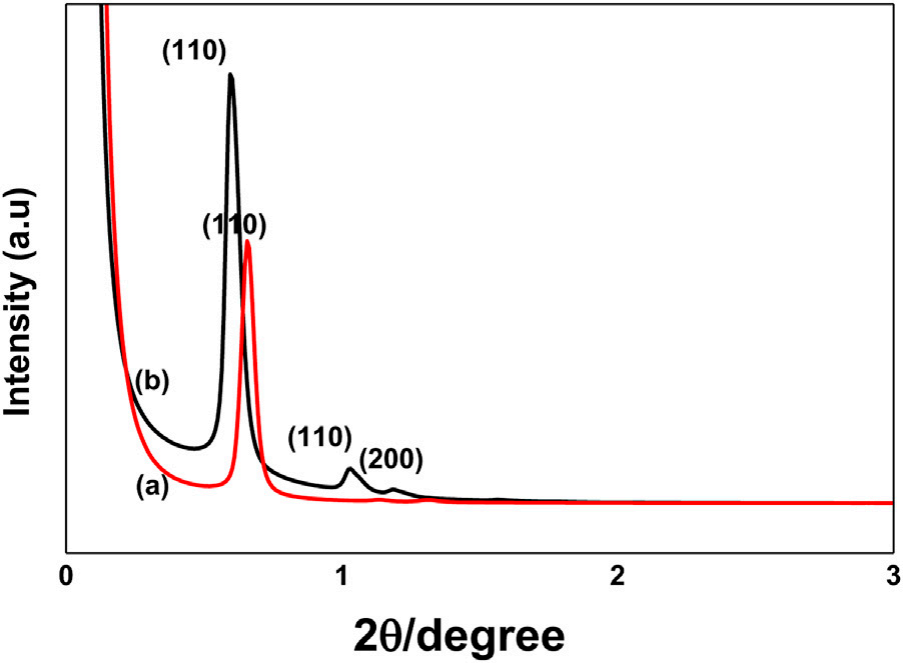
Small-angle x-ray diffractograms of (A) SBA@N and (B) SBA@3N silica materials.

SAX patterns for SBA-15 and modified materials SBA@N and SBA@3N are shown in Figure 5. A major reflection (110) and two small reflections indexed as (110) and (200) at 2θ were observed, which indicated a well-ordered mesoporous structure with 2D hexagonal p6mm symmetry as reported for this type of mesoporous silica material (Ahmed et al., 2016; Rehman et al., 2018). For both materials, the appearance of well-resolved diffraction peaks also indicates that surface modification did not affect the original structure of SBA-15.

### 4.6 SEM analysis

**FIGURE 6:**
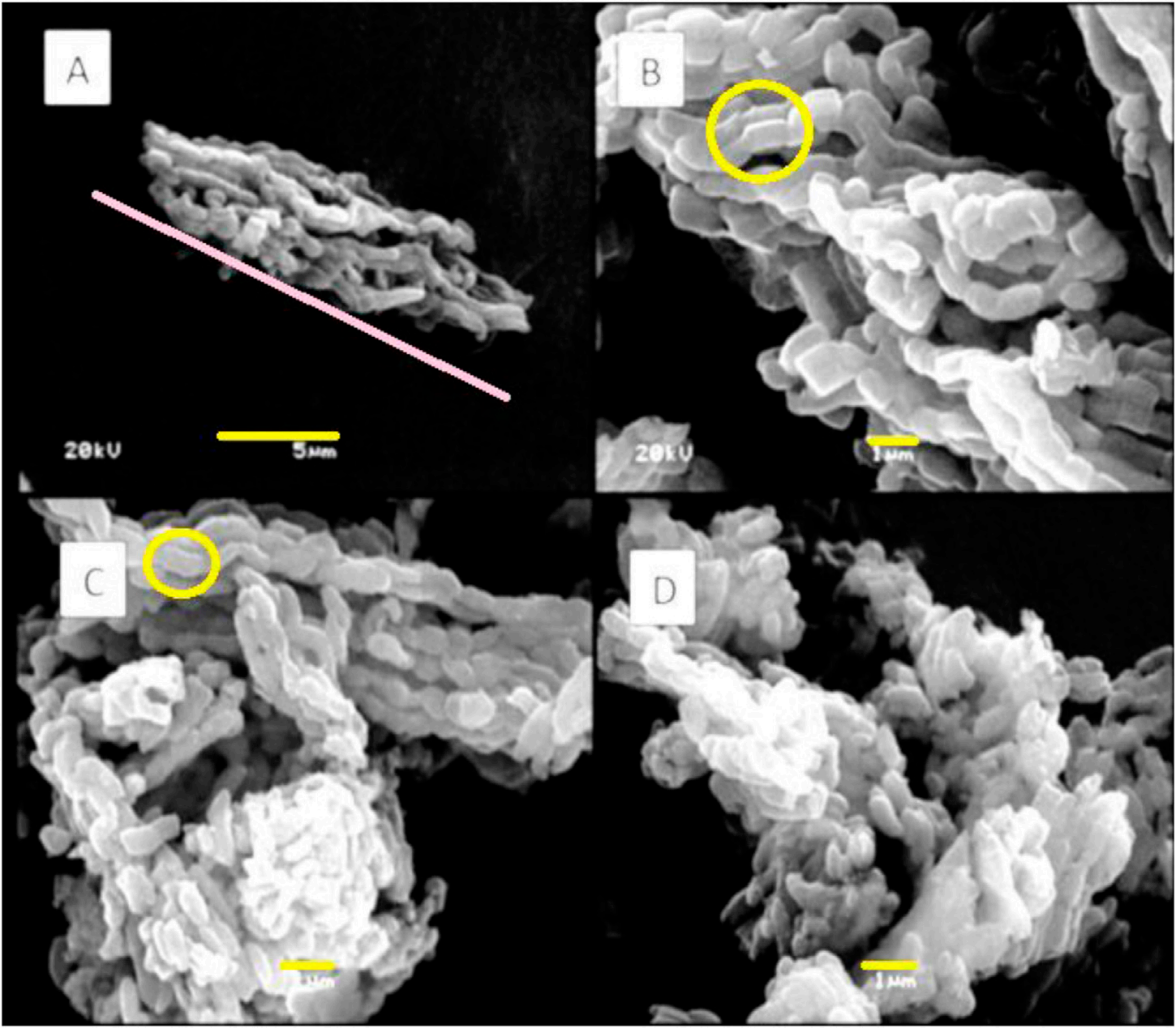
SEM micrographs of **(A,B)** SBA-15 with different magnifications **(C)** SBA@N and **(D)** SBA@3N, scale bar 1 μm and 5 μm, and magnifications ×10,000 and ×3,500.

Scanning electron micrographs of the prepared materials are depicted in Figure 6. A typical morphological structure of SBA-15 with channel-like long fibers was observed for all materials as reported previously (Heidari et al., 2009; He and Shi, 2011; Tariq et al., 2019; Osman et al., 2020; Rahman and Nasir, 2020; Al-Gamal et al., 2021). The average length of these fibers was between 10 and 12 μm, with an average sub-particle length of 1.2 μm of a diameter of 0.6 μm (Figures 6A, B). The same morphology was observed for the derived surface-modified materials, SBA@N and SBA@3N. The average sub-particle length of about 1.2 μm can be seen in SEM micrographs in Figures 6C, D. These micrographs showed that the original structure of typical SBA-15 silica remained preserved after heat treatment during calcination and surface modification.

### 4.7 Zeta potential

To quantify the electric charges on prepared silica micron-sized particles, zeta potential (ζ) was measured (Janiak and Kofinas, 2007; Tsai et al., 2009; Rehman et al., 2018; Khan et al., 2020). For unmodified SBA-15 with free surface silanols, the ζ value appeared at approximately −20.0 mV. A change in this value was noted for the surface-modified materials. For SBA@N and SBA@3N, ζ values appeared as −25.0 and 10.0 mV, respectively. This change in the electric charges was expected from the surface-modified materials in which the silanol groups were replaced with diethanolamine functional groups. The particle size was also measured with a Zetasizer. The particle diameter was found to be approximately 2.4 μm and 2.9 μm for SBA@N and SBA@3N, respectively, which agrees with the SEM data.

### 4.8 Drug loading and release

The drug loading capacities (wt/wt%) of the modified materials were calculated and found to be 9.42% and 9.83% for SBA@N and SBA@3N, respectively. Unmodified SBA-15 loaded a very small quantity of 5-fluorouracil.

**FIGURE 7:**
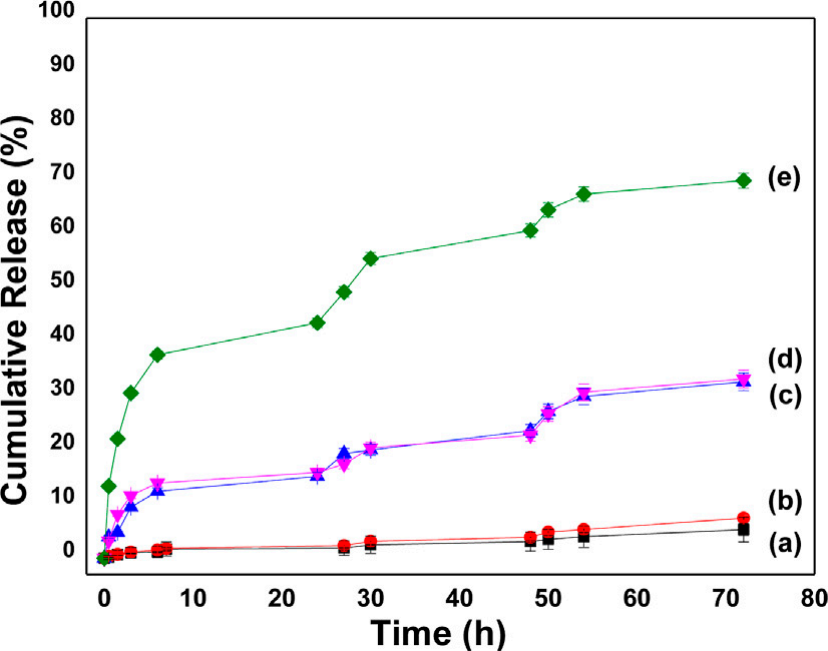
*In vitro* cumulative release profiles of 5-FU from (a)SBA@3N in SBF, (b)SBA@3N in SIF, (c)SBA@N in SIF, (d) SBA@N in SGF, and (e) SBA@N in SIF at 37°C ±1.

Simulated bodily fluid (SBF, pH 7.2, USP), simulated gastric fluid (SGF, pH 1.2 without pepsin, USP), and simulated intestinal fluid (SIF/phosphate buffer pH 6.8 without pancreatin, USP 26) were used to produce *in vitro* cumulative drug release profiles under sink circumstances at 37°C. Slow drug release kinetics was observed from the prepared materials; during the initial 6 h, less than 15% of 5-FU was liberated from the prepared carriers in all simulated physiological fluids (Figure 7). Material SBA@3N liberated approximately 8% of 5-FU in the SIF (pH 6.8) during 48 h. The release of 5-FU from SBA@3N into SBF (pH 7.2) was also slow. The material, SBA@N released about 60% of the total drug content within 48 h in SIF (pH 6.8), with an initial burst release of 13% (Figure 7). However, this material (SBA@N) released about 33% of 5-FU in SGF.

Compared to SBA@N, SBA@3N showed promising behavior, and a slow-release profile was observed in SBF and SIF, suggesting interactions of 5-FU with the functional organic groups. A clear difference in the drug release profile was observed from both materials. SBA@3N with a longer chain showed slow release of 5-FU, which indicates the significance of organic chain length attached to the silica surface *via* silanol chemistry. The longer this chain, the stronger will be the drug interaction, which results in a slow drug release process. In addition to these interactions, hydrogen bonding and other physiochemical properties such as small pore diameter, mesoporous network, and surface silanol are also the main parameters that contributed toward drug loading and release.

### 4.9 DFT studies

**FIGURE 8:**
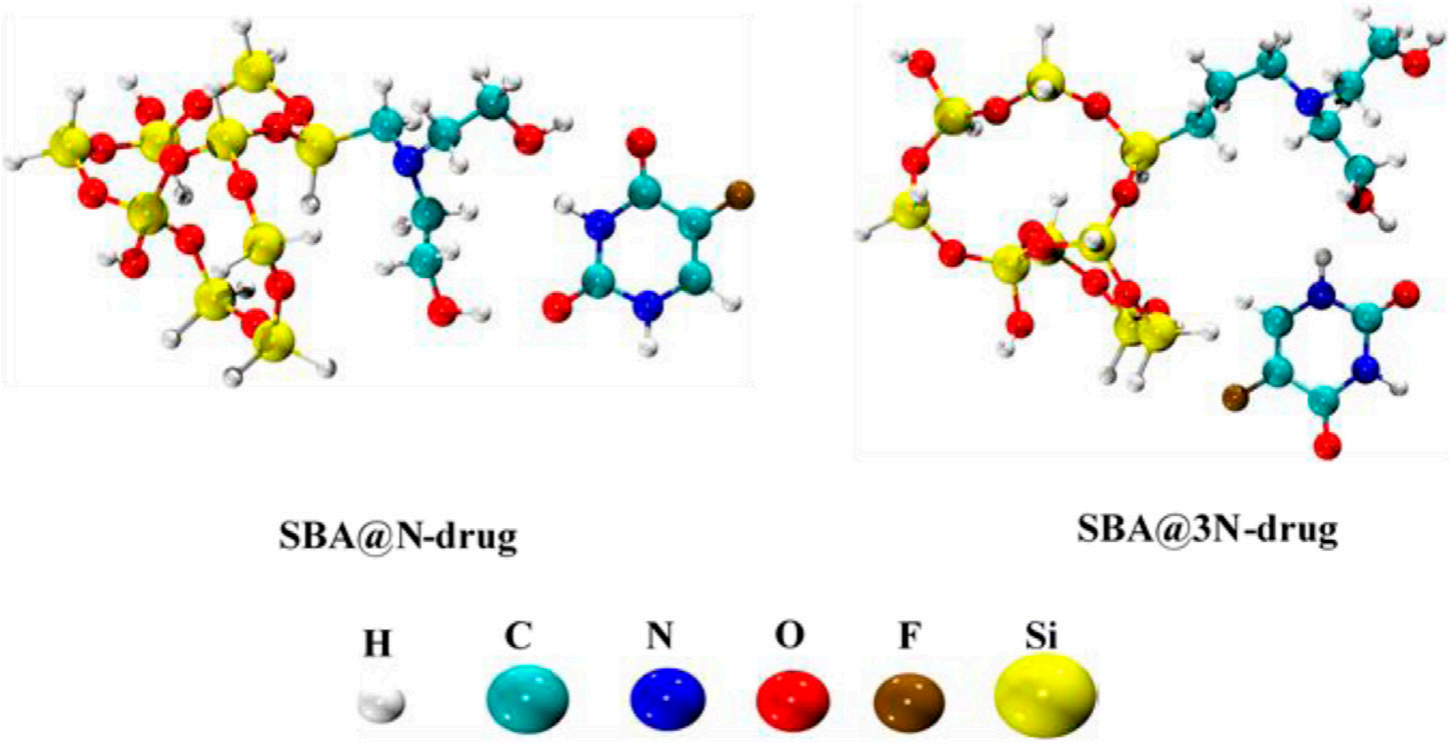
Optimized geometries of SBA@N–drug and SBA@3N–drug complexes.

Density functional theory (DFT) calculations have been performed to study the interaction of the 5-FU drug with both derivatives of SBA-15. A conformational analysis has been carried out for the installed side chains of the diethanolamine and the most stable geometries were selected for studying the interaction with the drug molecule. All interaction sites of 5-FU with SBA@N and SBA@3N have been considered, and the low-lying energy complexes (most stable ones) are shown in Figure 8.

It has been observed that hydrogen bonding is a decisive interaction factor in both the complexes (SBA@N–drug and SBA@3N–drug). The hydrogen bonds are formed between the carbonyl and amino groups from the 5-FU and the hydroxyl groups in the installed side chains of SBA@N and SBA@3N derivatives. The calculated interaction energies of both complexes can elucidate the strength of the complexes formed and their subsequent drug release. The interaction energy of the SBA@3N–drug (−17.5 kcal/mol) complex is 1.5 kcal more than that of the SBA@N–drug complex (−16.0 kcal/mol). The range of calculated energies indicates that the drug interacts with both derivatives of SBA-15 *via* physisorption. In addition, a slightly higher binding energy in the case of SBA@3N–drug indicates a slow release of the drug from the complex, which is in accordance with the experimentally observed results.

### 4.10 Drug release kinetics

Three parameters are used to assess the formulation’s drug release: 1) statistical method, 2) model-dependent method, and 3) model-independent method. In this work, we adopted the model-dependent method to evaluate the prepared formulations. The drug release data were processed with zero-order (ZO) and first-order (FO) kinetic models, Higuchi, Hixson–Crowell (H–C), and Korsmeyer–Peppas (K–P) models (Rehman et al., 2014a) (Eqs 7–12).

$$Zero-order\;model:\frac{mi}{mt}=kt,$$ (7)

$$First-order\;model:\ln\;\left(1-\frac{mi}{mt}\right)=-kt,$$ (8)

$$Higuchi\;model:\frac{mi}{mt}=k\sqrt{t},$$ (9)

$$Hixson–Crowell\;model:\sqrt[{3}]{\left(1-\frac{mi}{mt}\right)}=-kt,$$ (10)

$$Korsmeyer-Peppas\;model:\frac{mi}{mt}=n\;\mathrm{Int}+Ink,$$ (11)

$$non-linear\;Korsmeyer-Peppas:\frac{M_{t}}{M_{\infty }}=kt^{n},$$ (12)

where *mi/mt* are drug fractions released at time *t*; *n* is a diffusional exponent, which is an indicator of drug release mechanism; *k* is constant; *M*_*t*_ represents the mass of the drug liberated in the release medium at time t; and *M*_∞_ is the mass of the drug liberated at an infinite time.

The drug release model Higuchi is applied to planar, geometric, and porous systems. The Hixson–Crowell model is applied to systems where a change is expected in the surface area and diameter of particles or tablets, while the fitting of the K–P model describes the drug release mechanism, such as water migration into the matrix, matrix swelling, and matrix dissolution. This model explains the drug diffusion process in the polymeric and mesoporous material-based systems. The exponent value is *n* ≤ 0.45, suggesting that the drug is released from the system through Fickian diffusion (FD). The diffusional exponent values of 0.45 < *n* < 0.89 correspond to a non-Fickian or anomalous diffusion. The exponent *n* equal to 0.89 indicates case-II transport, while *n* > 0.89 represents typical ZO kinetics (Table 2).

**TABLE 2:** Drug transport processes and the diffusional exponent n, which specifies whether the drug diffusion is Fickian or non-Fickian from the release system.

Type of transport	Diffusional exponent(n)	Time dependence
Less Fickian diffusion	*n* > 0.45	
Fickian diffusion	*N* = 0.45	t/^1/2^
Non-Fickian (anomalous) diffusion	0.45 < *n* < 0.89	t^n−1^
Case II transport	*n* = 0.89	Time-independent
Super case II transport	*n* > 0.89	—

Eqs 7–12 were used to fit the release of drug data from the produced formulations, and the parameters obtained are listed in Table 3. The best fit of the models to the data released is shown by a high correlation coefficient (R^2^) value. A high *R* ^2^ value was observed with the K–P model. The FD mechanism of 5-FU in SGF and SBF was shown by the n value (0.45). The release of 5-FU in SIF from SBA@N and SBA@3N followed non-Fickian diffusion and Super case II transport and FO kinetics, respectively.

**TABLE 3:** Mathematical modeling of zero-order and first-order models to the drug release data up to 24 h and of Higuchi model, Hixson–Crowell (H–C), and Korsmeyer–Peppas (K–P) up to 6 h in simulated gastric fluid (SGF), body fluid (SGF), and intestinal fluid (SIF). The correlation coefficient (*R* ^2^), diffusion exponent (*n*), Fickian diffusion (FD), and zero-order (ZO).

Silica	Release medium	Linear fit				Non-linear fit		Mechanism	
		Zero-order	First-order	Higuchi	H–C	K–P		Diffusion	Kinetics
		*R* ^2^	*R* ^2^	*R* ^2^	*R* ^2^	*R* ^2^	n		
SBA@N	SGF	0.50	0.50	0.96	0.96	0.96	0.40	FD	ZO
SBA@N	SBF	0.13	0.50	0.98	0.98	0.98	0.46	FD	ZO
SBA@N	SIF	0.50	0.51	0.98	0.97	0.98	0.30	NFD	FO
SBA@3N	SBF	0.15	0.40	0.94	0.98	0.97	0.43	FD	ZO
SBA@3N	SIF	0.13	0.60	0.93	0.97	0.98	1.01	SC-II	ZO

## 5 Conclusion

This work reports the synthesis, surface modification, and characterization of mesoporous silica type SBA-15 and its novel derivatives SBA@N and SBA@3N. These materials were successfully synthesized and characterized with different techniques. The drug loading efficiency of these materials was ∼10%. Slow-release kinetics was observed in simulated fluids (SBF, SIF, and SGF), except for SBA@N in the simulated intestinal fluid. Different mathematical models predicted the drug release mechanism. The non-linear adjustment of the K–P model explains that the drug release is realized through Fickian diffusion, with zero-order kinetics. 5-FU drugs produce cardiotoxic byproducts that could be controlled by protecting drug molecules in porous channels in the basic intestinal media. When compared to SBA@N, SBA@3N showed promising behavior by showing a slow-release profile in all simulated fluids and suggesting strong interactions of 5-FU with the organic functional groups. This strong interaction of the 5-FU drug and hence its slow release has been supported by the interaction energies calculated from DFT simulations. Based on the results, we suggest that our prepared materials could be biocompatible carriers for short-life hydrophilic anticancer drugs, such as 5-fluorouracil, to overcome the shortcomings, such as shorter life in blood plasma and fast metabolism in the gastrointestinal tract. In future, *in vitro* and *in vivo* cytotoxicity experiments will be performed to elaborate on this work further.

## Data Availability

The raw data supporting the conclusion of this article will be made available by the authors without undue reservation.
